# Comparative architecture of the tessellated boxfish (Ostracioidea) carapace

**DOI:** 10.1038/s42003-024-07119-z

**Published:** 2024-11-26

**Authors:** Lennart Eigen, Jan Wölfer, Daniel Baum, Mai-Lee Van Le, Daniel Werner, Mason N. Dean, John A. Nyakatura

**Affiliations:** 1 Institut für Biologie, Humboldt-Universität zu Berlin, Vergleichende Zoologie, Philippstraße 13, 10115 Berlin, Germany; 2 Bernstein Center for Computational Neuroscience Berlin, Humboldt-Universität zu Berlin, Philippstraße 13, 10115 Berlin, Germany; 3 Zuse-Institut Berlin, Takustraße 7, 14195 Berlin, Germany; 4 Max Planck Institute of Colloids and Interfaces, Department of Biomaterials, Am Mühlenberg 1, 14424 Potsdam, Germany; 5 Present Address: Department of Infectious Disease and Public Health, City University of Hong Kong, Kowloon Tong, Hong Kong; 6 Centre for Nature-Inspired Engineering, City University of Hong Kong, Kowloon Tong, Hong Kong

**Keywords:** Ichthyology, Biomechanics

## Abstract

Tessellations (surface architectures of arrays of hard tiles) are common in natural and man-made designs. Boxfishes (Ostracioidea) are almost completely encased in a tessellated armor and have evolved a plethora of cross-sectional carapace shapes, yet whether the scutes constructing these exhibit comparable variation is unknown. Using high-resolution microCT and semi-automatic segmentation algorithms, we quantitatively examined thousands of scutes from 13 species of diverse body form. A cluster analysis revealed that certain scute types are associated with specific carapace regions independent of carapace shape. Scute types differentiate between carapace edges and flat regions, as well as between the head region with many carapace openings and the more consistently closed abdominal region, pointing at a constructional commonality or constraint shared by all boxfish species. However, the dimensions of edge scutes varied systematically with carapace shape (e.g., scute aspect ratio tended to increase with decreasing carapace height). This suggests that protection is maintained across body forms by managing scute- and carapace-level mechanisms for increasing bending resistance. Future studies on other taxa are necessary to understand whether these architectural principles are specific evolutionary solutions for building a boxfish carapace or whether they are shared by other biological systems that serve a similar protective function.

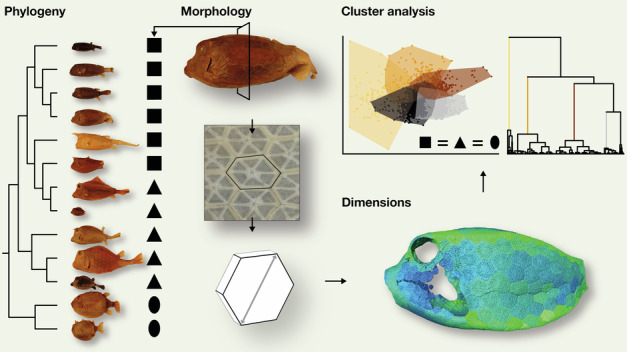

## Abstract (web summary)

Comparative architecture of the tessellated boxfish (Ostracioidea) carapace, using high-resolution microfocus computed tomography (microCT) and semi-automatic segmentation algorithms

## Introduction

A common tessellation pattern in both natural and man-made designs involves abutting, but non-overlapping tiles (i.e., positioned edge-to-edge). Hexagons (one of the only three regular Euclidean tilings, besides triangles and squares) are optimal for tiling flat surfaces in terms of tile density and mechanical robustness^1^. However, more convoluted and/or closed structures (e.g., spheroids) cannot be covered by hexagons only, necessitating the addition of other polygon shapes, a common example being the pentagons added in otherwise hexagonal patterns of classic soccer balls^2,3^. The possible tile geometries in such tessellations are thus constrained by the contours of the surface they cover and appear to follow principles of space optimization. Since organismic surfaces are often partly or completely closed, it is intriguing to examine how nature solves the topological problem of tiling diverse structures, particularly when other constraints are involved (e.g., mechanical demands or phylogenetic constraints on tile shape/size), as these natural solutions can also be leveraged for multifunctional bio-inspired designs.

**Fig. 1:**
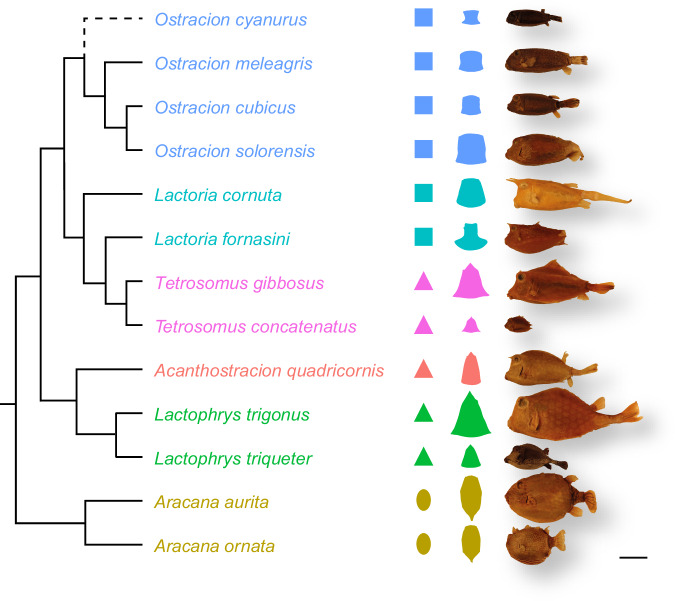
Phylogeny of Ostracioidea with studied specimens and cross-sectional shapes. Phylogeny adapted and modified from hypothesized phylogeny of Santini et al. ^6^ (note that *Lactoria* is paraphyletic), which did not include *O. cyanurus*. We included this species as the sister group of all other species of *Ostracion*. The dashed line indicates the uncertainty of its position. For simplicity, the phylogeny shows topological, but no distance information. Colored silhouettes illustrate simplified (left) and actual (right) cross-sectional geometries. The actual cross-sectional shapes were generated using surface models that were extracted from microCT scans. Each specimen is shown from the lateral view. Scale bar, 50 mm.

Boxfishes (Ostracioidea, Tetraodontiformes) are an ideal example for exploring natural tessellated architectures, being almost completely encased in a stiff tessellated carapace of comparatively large (i.e., easily visualized), interlocking, and calcified plates, called scutes^4–8^ (Fig. 1). The primary function of the stiff carapace is thought to be anti-predatory protection^9^, while the tessellated scute pattern prevents cracks from propagating through the carapace^10^. Despite the constraint of a closed covering of geometric scutes, boxfishes evolved disparate body cross-sections, from ellipsoid to triangular, rectangular to pentangular^6,7,11,12^ (Fig. 1), which likely reflect different evolutionary strategies for mechanical or hydrodynamic optimization^13,14^.

Eigen et al. ^8^ provided the first insights into how the boxfish carapace tessellation changes with animal size in an ontogenetic series of longhorn cowfish (*Lactoria cornuta*) (i.e., a tessellation altered to cover a dynamically changing surface). They found that scute dimensions mostly follow isometric scaling trajectories with increase of body size. For example, the relative area and relative maximum width of the scutes did not change despite a 10-fold increase in body length and hence, scute number also remained fairly constant. Furthermore, at all studied ages, the scutes were thickest and largest in volume along the edges (i.e., the weak points in a box-like architecture), while scute area and width were largest along the flat sides of the carapace. Scutes along the edges displayed the only substantial deviations from isometry by thickening relatively slower than expected under isometry, while the thickness of the scutes of the flat regions of the carapace scaled isometrically. This suggests the factors driving tessellation pattern evolution may vary on flat versus cornered surfaces^8^. The tiling pattern being consistent across ontogeny in the longhorn cowfish on the one hand and regional adjustments of tile thickness on the other allow maintaining the ability of the carapace to grow mainly proportionally through simple tile enlargement in flat regions, while also conserving the carapace’s overall protective function at all sizes^8^. However, during ontogeny, the carapace shape of the longhorn cowfish only undergoes a relative anteroposterior elongation and a minimal cross-sectional shape change^8^.

In comparison, the interspecific disparity in carapace cross-sectional shapes across the boxfish species represents an unexplored level of architectural complexity, i.e., varying in number and dominance of flat sides versus edges. One would expect this diversity to be accompanied by much more substantial adjustments in tessellation architecture among species.

Here, we use the boxfish carapace to understand how the shape of a tessellated surface affects its tessellation characteristics, investigating a sample of 13 species representing all major lineages within the group covering a four-fold range in body length and capturing the major boxfish body shapes (Fig. 1). By employing high-resolution microCT scans and a semi-automated segmentation algorithm, we quantify and visualize the characteristics of the hundreds of scutes that build up the carapace of each species, exploring how the diverse architectures maintain functional integrity while accounting for differences in relative body size and shape in a comparative phylogenetic framework.

## Results

### Carapace shape diversity

**Fig. 2:**
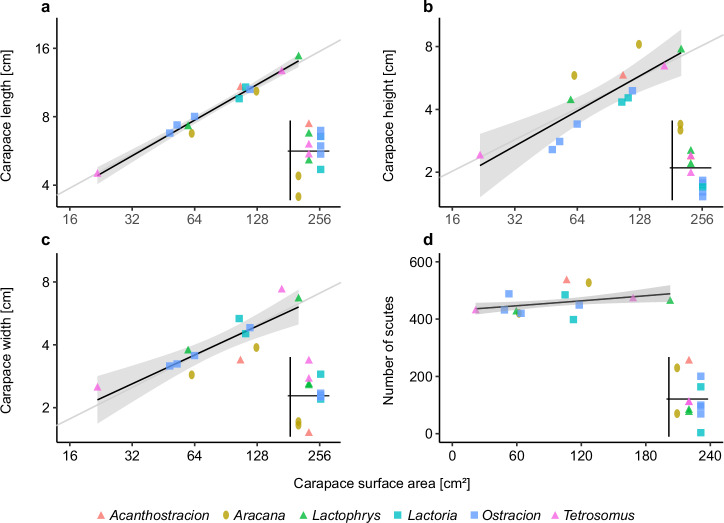
Dimensions of carapace and number of scutes against carapace surface area in Ostracioidea. **a** Carapace length. **b** Carapace height. **c** Carapace width. **d** Number of scutes. Log-log-scales were used for (**a**–**c**). The shaded area represents the 95% confidence interval of the model parameters from a linear regression model (**a**–**c**) or a Poisson regression model with a log-link function (**d**). The grey line represents isometric scaling in (**a**–**c**). The small plots in the right corner of each panel represent the residuals separated along the *x*-axis by simplified carapace geometry for easier comparison.

Carapace shapes have been traditionally described according to their cross-sections, since features like edges and flat sides do not change significantly along the anteroposterior body axis (see Fig. 1). Indeed, although all carapace dimensions (i.e., height, width, and length) scaled isometrically with carapace surface area (SA, which we used as a body size variable throughout this study), the confidence interval of carapace length was smallest compared to width and height (Table 1). It can also be seen from the spread of the residuals in Fig. 2 that the variation for a given SA was smallest for carapace length. For comparisons of boxfish carapace dimensions and scute variables, we categorized our sample into simplified carapace cross-sectional geometries, i.e., elliptic (*Aracana*), triangular (*Acanthostracion*, *Lactophrys* and *Tetrosomus*), and tetragonal (*Ostracion* and *Lactoria*) (Fig. 1). Note that, for simplicity, we characterized the carapace shapes of *O. cyanurus* and *L. fornasini* as tetragonal despite them having strongly concave lateral sides (Fig. 1).

**Table 1:** Regression results of interspecific scaling analyses against carapace surface area

		Slope	95% Confidence Interval
Carapace dimensions			
Length		0.52	0.46, 0.60
Height		0.56	0.32, 0.80
Width		0.46	0.28, 0.64
Scute number		1.00	1.00, 1.00
Scute curvature			
Curvature Gaussian surface		−1.16	−1.73, −0.58
Curvature mean surface		−0.48	−0.71, −0.26
Scute dimensions			
Volume	flat region	1.19	0.93, 1.44
	edge region	1.11	0.58, 1.64
	both regions	1.15	0.88, 1.41
Area	flat region	0.92	0.78, 1.06
	edge region	0.95	0.64, 1.26
	both regions	0.93	0.7, 1.17
Width	flat region	0.45	0.39, 0.51
	edge region	0.41	0.28, 0.55
	both regions	0.43	0.36, 0.5
Thickness	flat region	0.36	0.23, 0.49
	edge region	0.37	0.14, 0.59
	both regions	0.36	0.2, 0.53
Aspect ratio	flat region	−0.15	−0.25, −0.04
	edge region	−0.12	−0.23, −0.02
	both regions	−0.14	−0.3, 0.02

All regression coefficients except for scute number were obtained from a linear regression of log-transformed variables. The median was used for the variables associated with scute curvature and scute dimensions. Scute number was regressed using a generalized linear regression with a Poisson distribution and a log-link function and the slope was back-transformed to the response scale using the exponential function. Note that its confidence interval was very small and is not recognisable due to rounding. *N* = 13 for all statistical analyses.

Carapace cross-sectional shape was not related to body size since all simplified geometries were present in small and large species (Figs. 1 and 2). Also, all carapace dimensions scaled isometrically with body size (Table 1). However, when correcting for body size (i.e., by just considering the distribution of regression residuals) carapace dimensions showed specific trends. For example, the ellipsoid carapaces of *Aracana* were particularly high (dorsoventrally) but narrow (mediolaterally) compared to the triangular and tetragonal species (Fig. 2b, c). Triangular carapace shapes differed from tetragonal ones in being relatively higher despite otherwise having similar widths (Fig. 2b, c).

### Effect of carapace size and shape on scute number, scute shape, and scute dimensions

**Fig. 3:**
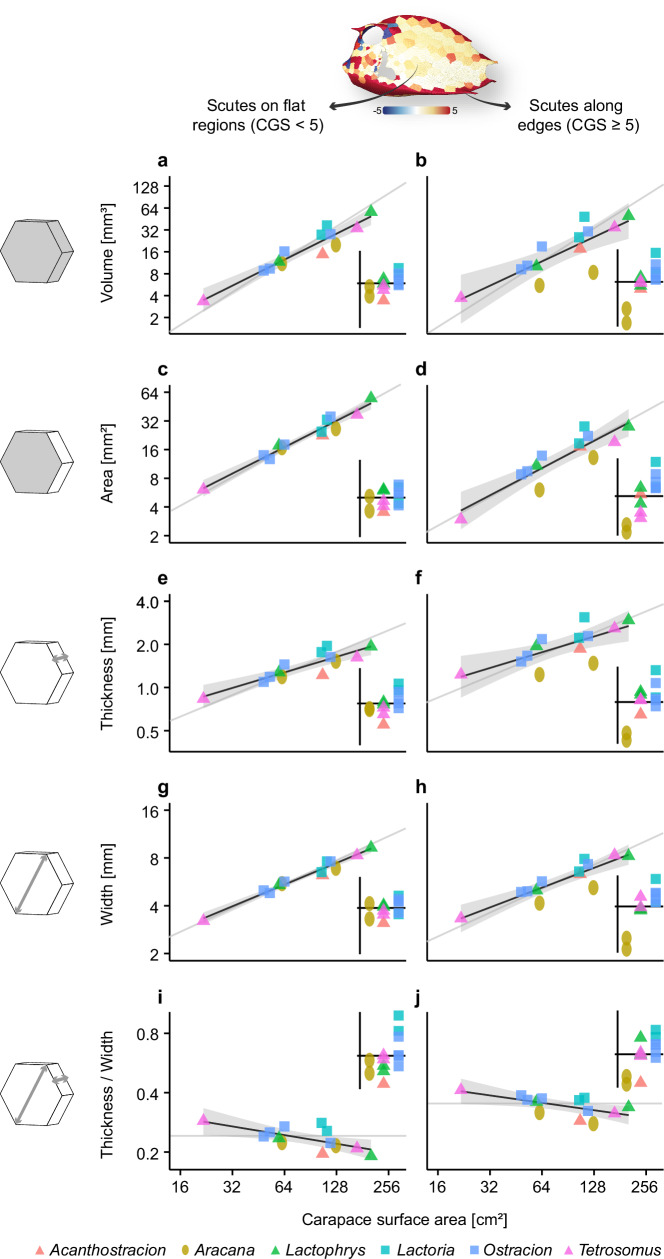
Regression plots of scute dimensions against carapace surface area for studied species of Ostracioidea separated by flat regions and edges. Flat regions and edges were defined according to the Gaussian surface curvature (CGS) of the scute. Median values of scute dimensions (illustrated by 3D icons) regressed on carapace surface area. Log–log-scales were used. The grey line represents isometric scaling. The shaded areas represent the 95% confidence interval of the model parameters from a linear regression model. The small plots in the right corner of each panel represent the residuals separated along the x-axis by simplified carapace geometry for easier comparison.

**Fig. 4:**
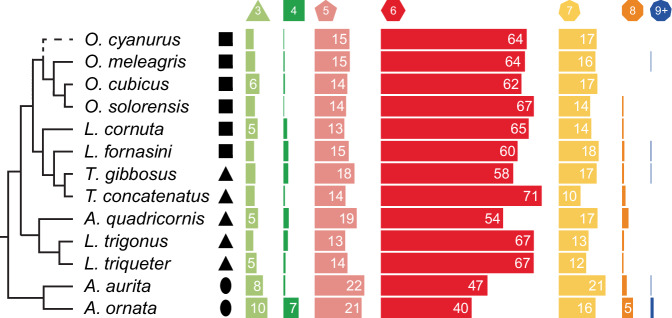
Frequency of scute shapes for studied species of Ostracioidea. Black silhouettes represent simplified cross-sectional geometries. Numbers only shown for frequencies ≥5.

**Fig. 5:**
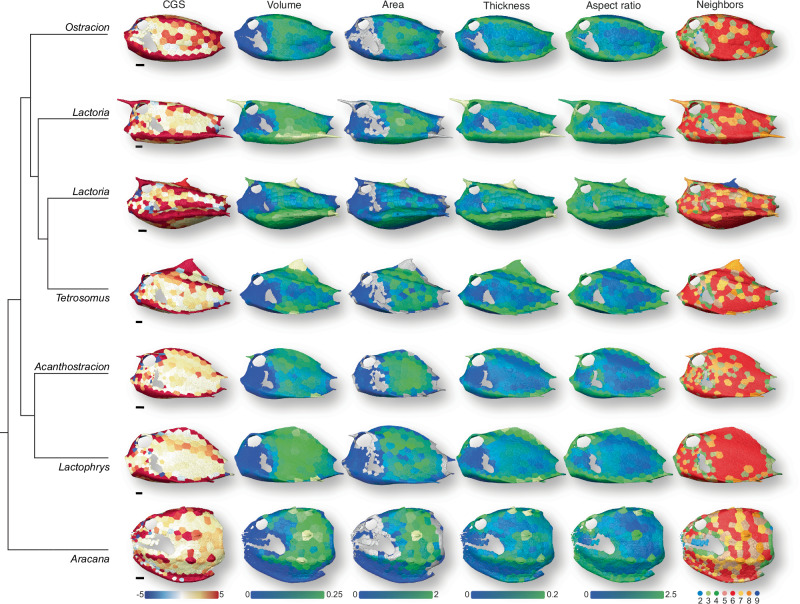
Surface renderings of boxfish carapaces with scutes color-coded according to different normalized variables and number of neighbors. One species per genus is shown from the left lateral view (note that both species of *Lactoria* are shown because they are paraphyletic). *Ostracion* (*O. solorensis*), *Lactoria* (*L. cornuta* above *L. fornasini*), *Tetrosomus* (*T. gibbosus*), *Acanthostracion* (*A. quadricornis*), *Lactophrys* (*L. trigonus*), *Aracana* (*A. aurita*). The grey scutes around the carapace openings represent cases in which the area could not be computed. Scute colors might fall outside of the color range (e.g., yellow scutes in the volume column that have a larger value than 0.25). This was necessary to optimize the visibility of the color gradient for all specimens and to highlight outliers (e.g., scutes with horns and other ornamentations). CGS Gaussian surface curvature. Scale bars, 10 mm. See Fig. S1 for renderings of mean surface curvature and scute width.

We regressed the number of scutes within a carapace as well as different scute dimensions (volume, area, thickness, width, and aspect ratio = thickness/width) onto SA and extracted the residuals to specifically focus on differences that we could relate to carapace shape. For scute dimensions, we conducted separate analyses on scutes belonging to flat regions and edges of the carapace (Fig. 3), since these areas differed from a morphofunctional perspective in a previous boxfish study^8^. Scute shape was quantified by identifying the number of neighboring scutes, for example, a scute with six neighbors was described as being hexagonal. However, this correspondence did not apply to scutes surrounding carapace openings, because these scutes usually had at least one edge without a neighbor. Thus, we calculated the proportion of scute shapes of each specimen excluding opening scutes (Fig. 4). Besides these statistical trends, we also color-coded the scute renderings of the carapaces according to the analyzed parameters so that we could assess how the tessellation properties were distributed across the carapace and if this was related to carapace shape (Fig. 5).

The number of scutes increased very slightly with SA (Fig. 2d, Table 1), but the fact that the minimum and the maximum number of scutes were both observed in medium-sized specimens indicates that body size (SA) does not affect the number of scutes in boxfishes (Fig. 2d). There was also no effect of carapace shape on the distribution of the residuals, i.e., all three carapace shape types had specimens above and below the regression line.

When looking at the distribution of the scute characteristics across the carapace, regional trends become apparent that justify the distinction between flat regions and edges (Fig. 5, Fig. S1). The edge scutes are distinctive in having large Gaussian and mean curvature values (CGS and CMS, respectively; Fig. 5, Fig. S1). They are consistently thicker and as a result have a higher aspect ratio (~0.3–0.4 compared to ~0.2–0.3) than scutes from flat regions, while the latter have larger areas (Fig. 5). Only the *Aracana* specimens deviate from this pattern in possessing edge scutes that are smaller in all ways than scutes from flat regions, in terms of median values (volume, area, thickness and width; Fig. 3). According to Fig. 5 and S1, these differences are related to the large keel that constitutes the ventral edge of *Aracana*’s carapace. Also, a few species (e.g., of the genera *Aracana* and *Lactoria*) possessed scutes with outlying values that relate to the existence of horns and ornamentations. Scutes that form horns and ornamentations were relatively thicker and more voluminous compared to their neighboring scutes as seen particularly in species of *Aracana*, but also of *Lactoria* and *Tetrosomus* (Fig. 5, Fig. S1).

The relative frequencies of scute shapes were very consistent across all studied species despite the observed disparity in carapace size and shape. Hexagonal scutes were the most frequent in all carapaces (40–71%), followed by pentagonal (13–22%), heptagonal (10–21%), triangular (3–10%), and finally tetragonal (0–7%) and octagonal ones (0–5%) (Fig. 4). Some specimens also had a few scutes with more than eight neighbors. Only *Aracana ornata* stood out in having comparably many scutes with small or large numbers of neighbors (Fig. 4). Tile shape (i.e., number of neighbors) appeared to be randomly distributed across body regions, though some regions in some specimens were consistently covered by large batches of hexagonal scutes (e.g., continuous red regions in Fig. 5). However, the presence of such batches was not related to carapace shape, size, or phylogenetic affinity.

All scute dimensions scaled isometrically with body size independent of whether they belonged to the flat region of the carapace or its edges (Fig. 3, Table 1). However, the aspect ratio scaled with slight negative allometry (Fig. 3i, j, Table 1). When looking at the regression residuals, the effect of carapace shape on the scute dimension was much stronger in the scutes from the carapace edges than those from the flat region (Fig. 3). The median values of all dimensions of edge scutes tended to be largest in boxfishes with tetragonal shapes, intermediate in triangular ones, and smallest in ellipsoid boxfishes (Fig. 3).

### Clustering and covariation of scute characteristics

We applied a more in-depth quantitative approach to gain further insight into the architectural patterns of the carapaces beyond the simple distinction between flat and edge regions. Firstly, we conducted a correlation analysis (pairwise scatterplots and Pearson's correlation coefficients as well as a principal component analysis (PCA)) using all scutes of all specimens to understand the interrelation of scute characteristics. These were scute thickness, width, area, volume, aspect ratio, number of neighboring scutes as well as local curvature measures CGS and CMS (all variables were size corrected and log-transformed). We also used a hierarchical tree cluster analysis to explore whether scutes form clusters that could be attributed to carapace shape or phylogeny.

**Fig. 6:**
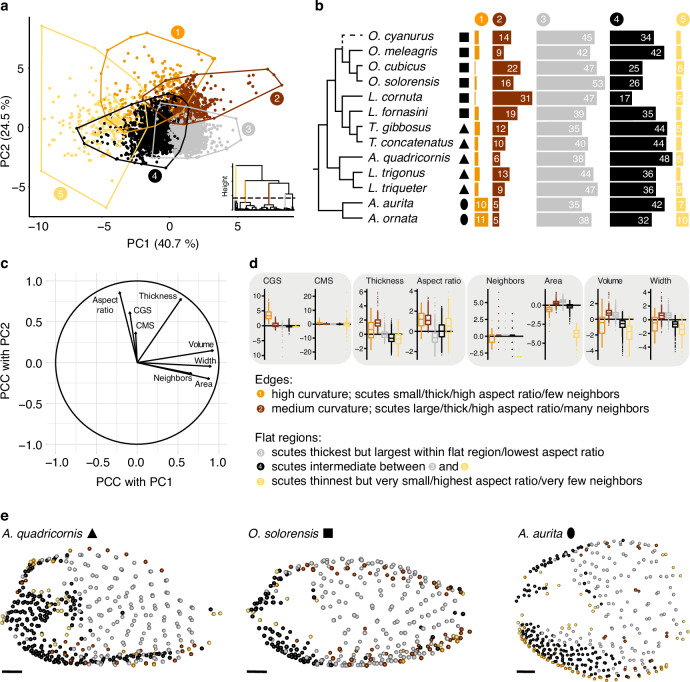
Results of the correlation and cluster analysis of scute variables. **a** First two principal components (PC) with point clusters. Prior to PCA, the variables were transformed (i.e., size-corrected, log-transformed and finally standardized (centered and scaled)). Each convex polygon belongs to one numbered cluster. The hierarchical tree of the cluster analysis is shown in the bottom right with the first branch of each cluster color-coded. **b** Frequency of each cluster for each specimen. Numbers are only shown for frequencies ≥5. Black silhouettes represent simplified cross-sectional geometries. **c** Loading of variables onto principal components as indicated by the Pearson’s correlation coefficient (PCC). **d** Cluster-specific boxplots for each scute variable (variables were size-corrected, log-transformed, and standardized as prior to PCA, see (**a**)). The variables were grouped (grey boxes) according to similar clustering patterns. CGS Gaussian surface curvature, CMS mean surface curvature. **e** Lateral view onto scute coordinates of an exemplary specimen from each simplified cross-sectional geometry colored according to cluster association (**a**). Scale bars, 10 mm.

When looking at the two first PCs (explaining ~66% of variance), it becomes apparent that all species occupy the same region of the scute morphospace, indicating that the variability of scute characteristics is very similar in all studied species independent of carapace size, carapace shape and phylogeny (Fig. S2a). Instead, we identified clusters that partitioned scute variability among five major scute types (Fig. 6a). These clusters relate to different regions of the carapace and refine the edge versus flat region dichotomy. Clusters 1 and 2 discriminated scutes from the edge regions, whereas clusters 3 to 5 divided the flat regions into specialized subregions. Within edge regions, scutes from cluster 1 are specifically located at areas with the highest curvature, are smaller in all morphological descriptors (except for aspect ratio, which is similar) and have fewer neighbors compared to scutes from cluster 2 (Fig. 6b, d, e).

Within the flat regions a certain trend is apparent from clusters 3 to 5. Scutes from cluster 3 cover the abdomen; they are largest in all dimensions compared to the other two clusters, but have the smallest aspect ratio. Scutes from cluster 5 surround carapace openings and are smallest in all dimensions, but have the largest aspect ratio. In addition, they have the fewest neighbors of all five clusters. The scutes from cluster 2 fall between these two clusters and cover the head and the pectoral region and sometimes extend more or less onto the abdomen (Fig. 6).

This comparison of cluster characteristics illustrates that the variables describing scute morphology displayed different patterns of covariation. On the one hand, in certain variables, covariation was manifested globally across all scutes independent of cluster association. For example, volume and width were strongly correlated linearly as visible in Fig. S3 and as indicated by the loadings in Fig. 6c, and both variables showed a similar trend across all five clusters (Fig. 6d). Number of neighbors and area also displayed a very similar pattern of discrimination between clusters. On the other hand, some variables only covaried in specific carapace regions. For example, as described above, scute thickness, volume and width were positively correlated, but negatively correlated with aspect ratio across the three clusters of the flat carapace regions (Fig. 6d).

While all specimens shared the same scute differentiation revealed by the cluster analysis, the frequency of the scutes of each cluster did vary among species (Fig. 6b). For example, both specimens of the genus *Aracana* had particularly abundant scutes of clusters 1 and 5. Note, though, that the relative frequency of the scutes in cluster 5 is biased downward, since area could not be computed for many of the scutes that surround carapace openings and we had to remove them in our multivariate analyses (see Methods and Fig. 5). Scutes from cluster 2 were distributed prominently along the ventral keel of *Aracana* (Fig. 6e, S2b). Otherwise, we could not identify any particular pattern of relative cluster frequency associated with carapace shape (Fig. 6b).

## Discussion

It has been suggested that the primary function of the rigid boxfish carapace is protection against predators^9,12^, while the evolution of different carapace shapes was likely driven by hydrodynamic optimization^13,14^. Here, however, we have demonstrated that, despite being shaped differently and spanning a large range of body sizes, the carapace tessellation in boxfishes is realized by a relatively consistent array of scute types. While Eigen et al.^8^ identified distinct morphological differences between scutes of the edges and the flat regions of the carapace in the boxfish *Lactoria cornuta*, we could identify a more fine-grained discrimination of five types of scutes. Each scute type, as identified by the cluster analysis, was associated with a specific regional topography of the carapace: (i) highly curved edges, (ii) moderately curved edges, (iii) flat sides of the abdomen, (iv) flat sides of the head and pectoral girdle, (v) and flat areas around the carapace openings. While a certain degree of overlap between the clusters exists (Fig. 6a), their strong topographical association supports the architectural relevance of the established clusters. Given that the origin of the group is ~63 million years ago (Santini et al.^6^), the surprisingly limited variation in these principal building blocks suggests that carapace construction is highly constrained either by a function essential to all boxfishes or through an inherited developmental program. This represents an exciting foundation for future evodevo work, since the robust link between scute morpho-class and local carapace contours suggests that whatever developmental processes distinguish edge and flat regions are major drivers in the development of scute type and therefore the evolution of boxfish carapace diversity.

The distinction between the two scute types along the edges is often based on whether a scute sits directly on top of the edge (i.e., the region of highest curvature) or whether it is associated more peripherally with an edge (moderate curvature, Fig. 6e, Fig. S2b). Interestingly, although the scutes associated with high carapace curvature are smaller in all dimensions compared to those associated with moderate curvature, both scute types share a similar aspect ratio. This hints at potential developmental and constructional constraints for edges, that edge scutes might vary in their overall dimensions, but exhibit relatively consistent shape characteristics.

The establishment of different scute types in the flat carapace regions according to the three clusters can likely be explained by constructional constraints involved in shaping fine-sculpted surfaces. The head and pectoral regions, for example, are especially structurally complex, perforated by carapace openings and having high local variation in surface curvature, although on average being comparatively flat. These irregular surfaces are covered with a mix of smaller diversely shaped scutes (e.g., *Aracana*, Fig. 5), indicating that more elaborate surface details are achieved by reducing and diversifying scutes to match the level of detail. In contrast, the flat abdomen was the only region where a single scute shape (hexagon) was found in high proportion across the surface. This particular regularity was also observed across ontogeny of *Lactoria cornuta*^8^. The otherwise irregular distribution of scute shapes across the carapace suggests that, compared to a soccer ball, for example, boxfish carapaces are overall too topographically complex to be tiled by just two scute shapes, although there is some consistency of tile shape in areas where local curvature is more invariant (Fig. 5). It remains to be shown how scutes form early in their development and change during ontogeny and why this relates to the diversity of different scute shapes across the carapace surface.

The carapace SA can either be composed of many small scutes or fewer larger scutes. When the carapace surface grows during ontogeny or becomes larger in evolution, either by isometric or allometric scaling (the latter due to a change in carapace shape), scute number and/or area could theoretically be altered to cover the larger surface. We could show that scute area and width scales isometrically with boxfish carapace surface area, while scute number remains fairly constant on average. Eigen et al.^8^ observed the same trend throughout the ontogeny of *L. cornuta*. However, both their intraspecific and our interspecific studies also found a certain degree of variation in the scute number and area, which was neither related to carapace size nor to carapace shape. It could be related to random temporal fluctuations in the activation/inhibition of the developmental pathway of the scutes across the carapace during ontogeny. However, interspecifically the number of scutes varies three times more than intraspecifically (398–535 versus 354-397 scutes; see Eigen et al.^8^). Thus, changes in boxfish carapace SA over macroevolutionary timescales seem to be related to changes in overall scute number, whereas increases in scute area are associated with body form changes during ontogeny, at least in *L. cornuta* (the latter mechanism also observed in the cartilage tesserae of sharks and rays^15,16^). Since carapace size and shape do not affect scute number, we assume that the large interspecific variation in the number of scutes is the result of random genetic drift and that the different constructional strategies observed represent similar biomechanical strategies for building the boxfish carapace surface. Modeling and performance testing of bio-realistic tilings, representing the range of natural scute pattern variation, would be valuable for testing these hypotheses^17^.

The lack of variation in the relative proportions of scute shapes across the studied species has also been shown in previous studies on boxfish tessellation^5,8,10^. In these, as well as in our study, hexagons were the most frequent tile shape, balanced by pentagons and heptagons as the second most frequent geometries. Other polygons with fewer or more edges were less frequent. Only the genus *Aracana*–a distinct lineage from the other studied species^6^–displayed slight deviations from this pattern. Incorporating more species from the Aracanidae that differ in carapace shape from *Aracana* in future studies could reveal whether this deviation is a phylogenetic character of this taxon, or whether this bears a functional relevance for ellipsoid body shapes. Independent of the (cross-sectional) shape of the surface, the proportions of pentagons and heptagons were very similar (Fig. 4), while a dominance of pentagons would be expected for (almost completely) closed, convex three-dimensional surfaces^18^. As discussed above, this deviation might be partly related to the openings of the head and pectoral region.

The primary purpose of a tessellated carapace is commonly reported as mechanical protection against predatory attacks, by minimizing the stress levels transmitted to the underlying soft tissue and vital organs^19–24^. Failure of a natural armor could lead to direct predation, but also to infection, as well as vulnerability in subsequent territorial fights, and eventually to death^25–27^.

The weak points in a box-like architecture are the edges and corners^28^. Indeed, Eigen et al.^8^ demonstrated that the edges are relatively thicker in smaller individuals as compared to larger individuals of *L. cornuta*, potentially accounting for the smaller body size when attacked by predators. Still, edge scutes were thicker than scutes from flat regions even in larger specimens, which may be associated with the need for increased bending resistance specifically in these carapace regions^8^. We observed similar patterns in our interspecific dataset. Scute aspect ratio was largest in smaller species (as indicated by the slight negative allometry) and always larger in edge regions, independent of body size (Fig. 3i, j).

Furthermore, changes in carapace shape appear to be a strong driver for adjustments of edge scute dimensions, which is not the case for flat regions of the carapace. In particular, there appears to be a clear trend between carapace height and scute aspect ratio (i.e., how thick scutes are in proportion to their in-plane dimensions). The ellipsoid carapaces of *Aracana* possess the lowest scute aspect ratio for their body size along the edges (Fig. 3j). The reduced potential to resist bending might be compensated by their dorsoventrally-extended cross-section and prominent ventral keel, which together increase the dorsoventral second moment of area (Fig. 1). However, the presence of additional spikes along the ventral keel, for example in *A. ornata*, might further lighten selective pressure on scute morphology to resist bending (Fig. 1). Indeed, their relative carapace height exceeds that of the other boxfish shapes (Fig. 2d). Similarly, *Acanthostracion* has the smallest aspect ratio among the boxfishes with triangular cross-sections but has one of the largest carapace heights after accounting for body size. In contrast, the genera *Lactoria* and *Ostracion* with their tetragonal shapes have the smallest carapace height and appear to compensate for increased bending stresses by having some of the largest scute aspect ratios. The triangular species of *Tetrosomus* and *Lactophrys* (*L.* *triqueter*, though not *L.* *trigonus*) fall in between, possessing intermediate carapace heights and scute aspect ratios. This suggests that protection is maintained across diverse body forms by balancing mechanical demands at different architectural scales, where evolutionary changes in carapace geometry that result in lower body flexural stiffness (i.e., decreased carapace height) are counteracted by edge scute morphologies that increase regional bending resistance. Expanding Yang et al’s.^10^ studies of scute mechanical properties to examine site-specific differences in performance (e.g., relative to scute geometry and aspect ratio) is necessary to frame scute-level contributions to carapace mechanics.

Marcroft^12^ showed that the possession of lateral keels increases the average stress experienced by the carapace for a given force when being compressed (i.e., bitten) dorsoventrally. This could further explain, for example, why *Tetrosomus* with its lateral keels has a similar relative scute aspect ratio as *Ostracion* despite having relatively higher carapaces, or why *Lactophrys trigonus* with its lateral keels has a larger relative scute aspect ratio than *L. triqueter* (see Figs. 2 and 3). A larger relative aspect ratio could then compensate for the increased stresses experienced by the lateral keels.

Besides its lower aspect ratio, *Aracana* also notably stands out in possessing numerous, outstandingly small scutes along its edges, especially along its ventral keel. Subdividing a surface into a larger number of elements will increase the overall number of interfaces, which represent structural discontinuities. Yang et al.^10^ demonstrated experimentally in *L. cornuta* that, when the carapace surface is penetrated, the majority of the stress is transferred to the collagen base of the scutes via the sutures between scutes, instead of to the mineralized plates themselves. The increased scute interface area in the ventral keel of *Aracana* could make the carapace of this species more prone to penetration and crack propagation compared to the other boxfish species. Similarly, the small and irregular scutes around carapace openings in all studied specimens could indicate that the regions are generally more prone to penetration (or more flexible) than the scutes of the flat regions which are extensively covered by larger, regular (mostly hexagonal) scutes, but more experimental examination is needed to confirm this. However, it remains unclear what loading scenarios are actually relevant for different regions of the boxfish skeleton. Marcroft^12^ applied finite element modeling to abstracted surface models to come to their conclusions. A combined analysis of scute dimensions and finite element modeling could further elucidate how different combinations of carapace shape and tessellation influence stress resistance of the boxfish carapace to predator biting. To make such models informative, we need to understand how the carapace is loaded during predation, yet even basic ecological information on predator-boxfish interactions remains surprisingly poorly known^12^.

## Conclusion

The evolutionary diversity of the boxfish carapace offers great insights into how nature solves the topological problem of tiling both flat and highly-curved surface, even as the overall 3D form (here the boxfish carapace surface) was changing dramatically during diversification of the group. While the principal architectural units of the boxfish carapace, i.e., the scute types across carapace regions, have not changed over large evolutionary timescales, adjustments in scute dimensions along the carapace edges, in particular a scute’s aspect ratio, appear to play a significant role in maintaining carapace rigidity. On the contrary, aspects such as the tile number and area, which determine whether the carapace surface comprises many small tiles or fewer larger ones, seem to be more variable and less constrained by carapace shape. Future studies on the tessellations of other taxa are necessary to understand whether these architectural principles are specific evolutionary solutions for building a boxfish carapace or whether they are shared by other biological systems that serve a similar protective function. Since tilings are common in natural armors^29^, but their geometries only rarely quantified, the diversity of biological systems represent a fertile palette for understanding form-function relationships in organismal biomechanics.

## Methods

### Experimental model and subject details

The boxfish specimens used for this analysis came from the fish collection of the MfN (Museum für Naturkunde zu Berlin, Germany). We included 13 of the 37 extant species with one specimen each, covering both extant families (Aracanidae and Ostraciidae) and half of all currently recognised genera. Species include *Acanthostracion quadricornis, Aracana aurita, Aracana ornata, Lactophrys trigonus, Lactophrys triqueter, Lactoria cornuta, Lactoria fornasini, Ostracion cubicus, Ostracion cyanurus, Ostracion meleagris, Ostracion solorensis, Tetrosomus concatenatus, and Tetrosomus gibbosus*. The specimens were preserved in alcohol and did not show obvious pathologies.

### CT data acquisition and segmentation

Samples were scanned at the Max-Planck Institute of Colloids and Interfaces in Potsdam-Golm (MPIKG, Germany) with an RX Solutions EASYTOM µCT scanner (RX Solutions, France). Scans for all samples were performed with helix scan sample rotation, at 100 kV source voltage, 150 µA source current, 15 W, and 333 ms exposure. A beam hardening correction algorithm was applied during image reconstruction. CT images were visualized and processed with an extended version of the Amira software (AmiraZIBEdition, 2022 and 2023, Zuse Institute Berlin, Germany). The close association of scutes did not allow a purely automatic threshold-based segmentation. Thus, we adapted the semi-automatic seed-based segmentation workflow for segmenting the carapace and individual scutes, as applied in the analysis of boxfish anatomy by Eigen et al.^8^. First, the carapace was segmented using the ‘Threshold’ module. Then, we manually removed portions that were not part of the carapace (i.e., axial skeleton, eyes, fins, gills) using a combination of the ‘Brush’ and ‘Lasso’ tool. We then placed landmarks onto each scute in a 3D surface visualization of the carapace, using the ‘Isosurface’ module, which are the seeds for the ‘Propagate Contours’ module, from which the subsequent segmentation propagates (i.e., one region per landmark). Manual refinement of segmentations was done using the ‘Split Labels by Separation Surface’ module to split undersegmented scutes (i.e., labels including >1 scute), and the ‘Pick and Merge Labels’ module to merge oversegmented scutes (i.e., scutes divided into multiple labels).

### Carapace area, carapace geometry, and number of scutes

To obtain a surface representation from the segmentation of the scutes, we start from the region-adjacency graph (RAG), where each node represents one scute, placed in its center, and each edge connects two neighboring scutes. This graph forms a triangulation where each triangle connects three scute centers that mutually touch each other. In order to obtain a surface patch for each scute that approximates the scute surface, the dual graph of this initial triangulation (RAG) is computed by placing one node in each triangle and connecting the new nodes that surround one node in the initial RAG. To achieve this, several modules in the AmiraZIBEdition were used. First, the ‘Spatial Graph To Surface’ module was used to compute the initial surface from the RAG. The ‘Fix Orientation’ module was then used to fix the orientation of the triangles. Next, the ‘Dual Surface’ module was used to create the dual surface, followed by the ‘Fix Orientation’ module to fix the orientation of the triangles again. Then, the triangles were refined three times with the ‘Surface Refine Edges’ module with subsequent smoothing using the ‘Smooth Surface’ module (Iterations: 10; Lambda: 0.7). Finally, we used the ‘Surface Area Volume’ module in AmiraZIBEdition to compute the carapace surface area (SA) of each boxfish from the generated surface from each species.

We used the frontal view of the surface renderings to classify the cross-sectional carapace shapes into simplified geometries. Due to the small sample size, we defined three categories (Fig. 1): oval (*Aracana*), triangular (*Acanthostracion*, *Lactophrys*, *Tetrosomus*), rectangular (*Lactoria*, *Ostracion*).

Carapace length, height, and width were determined from the coordinates of the scutes. For this purpose, we conducted a principal component analysis using the ‘prcomp’ function in R version 4.3.3^30^ on the *x*, *y*, and *z* coordinates of each boxfish separately. The first principal component always represented the anteroposterior axis, the second and third ones either dorsoventral or mediolateral axes, depending on the shape of the specimen. We then calculated the absolute distance between the minimum and maximum values along each principal component to obtain carapace length, height, and width.

### Scute property definitions

The ‘Create Tesserae Statistics’ module in the AmiraZIBEdition was used to extract and characterize a variety of variables describing the morphology of scutes and their local environment (see Eigen et al.^8^). Inputs to the module were the label field containing the segmentation of the scutes, the RAG derived from this label field, and a surface representing the carapace shape. The following variables were computed: Firstly, (1) *number of neighbors*: number of neighboring scutes, evaluated on the RAG, as described above. Secondly, we quantified various scute dimensions, such as (2) *scute volume*: number of voxels * volume of a voxel; (3) *scute plane-based area (what we refer to simply as scute area elsewhere)*: the projected area of a scute, calculated for each scute by placing nodes in the centers of triangles/quads that surround the vertex of each scute in the RAG. If the scute vertex was not completely surrounded by triangles or quads (e.g., it was bordering an opening in the carapace), then all plane-based measures were set to −1000 to remove that scute from this variable’s analysis; (4) *scute thickness*: shortest dimension of a cuboid enclosing the scute; (5) *(maximum) scute width*: largest principal dimension of the cuboid, perpendicular to the thickness dimension. (6) *scute aspect ratio*: scute thickness divided by scute width, which is an index of a scute’s bending resistance. Finally, in order to understand the contribution of each scute to surface curvature, we also analyzed the local carapace curvature around each scute using (7) *Gaussian surface curvature (CGS)*: the product of the two principal curvature values of the surface field at the location of the scute’s center; (8) *mean surface curvature (CMS)*: the mean of the two principal curvature values of the surface field at the location of the scute’s center. All raw data regarding scutes can be found in Supplementary Data S1.

### Statistics and reproducibility

We used the software R version 4.3.3^30^ for all statistical analyses. In addition, we used the packages readxl^31^, tidyverse^32^, egg^33^, GGally^34^, and rgl^35^ for data manipulation and visualization. SA was used as an indicator of carapace size in subsequent analyses.

We transformed all carapace dimensions using the natural logarithm and used the ‘lm’ function to regress length, height, and width on SA (*N* = 13). We extracted the regression slope (i.e., scaling exponent) and used the ‘confint’ function to calculate its 95% confidence interval. For the number of scutes, we used a generalized linear model with a Poisson distribution and a log-link function as implemented in the ‘glm’ function to regress it on SA (*N* = 13). Again, the ‘confint’ function was used to compute the 95% confidence interval of the regression slope. We then transformed the slope value, and the confidence boundary levels back to the response scale by applying the ‘exp’ function to the parameter values returned by the ‘glm’ function. To analyze how carapace shape affects the carapace dimensions and the number of scutes independent of body size, we used a graphical approach by extracting the residuals from these regressions and plotting them against the simplified carapace geometries (Fig. 2). We did not directly include carapace shape as an independent variable into the linear regression models to analyze its size-independent effect on these scute dimensions because of the small sample size.

To analyze the number of neighbors, we generated color-coded scute surface renderings in the AmiraZIBEdition to analyze their spatial distribution (Fig. 5). We quantified scute shape by identifying the number of neighboring scutes, for example, a scute with six neighbors was described as being hexagonal. However, this correspondence did not apply to scutes surrounding carapace openings, because these scutes usually had at least one edge without a neighbor. Thus, we calculated the proportion of scute shapes of each specimen excluding opening scutes and compared these proportions across specimens and simplified carapace geometries (Fig. 4).

Of the remaining scute variables, we first studied scute curvature variables to identify curved and flat regions to analyze scute dimensions of these regions separately. We generated color-coded surface renderings of scutes according to their CGS and CMS values which allowed us to distinguish between scutes that belong to flat regions of the carapace from those that belong to carapace edges (Fig. 5, Fig. S1). To ensure comparability between differently sized specimens, we normalized (i.e., size-corrected) CGS and CMS values. For this purpose, we computed the median CGS and CMS values per specimen and conducted a simple linear regression with SA as the independent variable (*N* = 13) using the ‘lm’ function in R. The median was used instead of the arithmetic mean because of several extreme values in some specimens associated with scutes of peculiar morphology (i.e., horns). We log-transformed all variables prior to regression to ensure linearity despite different dimensionalities. We then normalized all CGS and CMS values according to the estimated scaling coefficient (−1.16 and −0.48, respectively, see Table 1) to account for allometric scaling effects, i.e., we divided all CGS values by SA^−1.16^ and all CMS values by SA^−0.48^. After inspection, we decided that renderings of the normalized CGS variable are preferable for identifying regions of different curvature. We considered a CGS value of 5 to be a good threshold, i.e., scutes with a CGS ≥ 5 are associated with carapace edges, while smaller values represent flat regions (Fig. 3).

Based on this curvature analysis, we divided our dataset into two subsets, one with low curvature scutes and one including those with high curvature. Using scute volume, area, width, thickness, and aspect ratio, we conducted separate simple linear regressions onto SA (*N* = 13) with each dataset (Table 1). We assessed the effect of carapace shape on the median of the scute dimensions independent of carapace size by extracting the residuals from these regressions and plotting them against simplified carapace geometry (Fig. 3). As for the number of neighbors and the two curvature variables, we also generated color-coded scute surface renderings to analyze spatial variation of the normalized scute dimensions across the carapace. Since, for each scute dimension, the regression slopes of both carapace regions were always similar (Table 1), we decided to reconduct the regression with the complete dataset to obtain a single regression coefficient that we used to normalize all scute values for the surface renderings. To justify this, we statistically tested whether flat regions and carapace edges differ in terms of these scaling exponents by including into the regression an interaction effect between SA and a binary variable that coded for the carapace curvature (high or low) (*N* = 26). The *p*-value of the interaction effect was 0.78 for scute volume, 0.84 for scute area, 0.57 for scute width, 0.97 for scute thickness, and 0.75 for scute aspect ratio. Since the interaction effect was never significant (alpha = 0.05), we decided that the scaling exponent is independent of carapace region. Thus, we used a single scaling exponent to normalize each scute dimension (Table 1) that we used to color-code the surface renderings (Fig. 5, Fig. S1).

For the covariation and cluster analysis, we first removed all scutes from the complete dataset for which the area could not be computed or was determined to be zero by the AmiraZIBEdition algorithm (see results). We included the number of neighbors, the normalized scute dimensions, and the normalized scute curvature variables. Again, we used the normalized variables to explore size-independent patterns. Then, all variables excluding the number of neighbors were transformed using the natural logarithm to linearize power relationships between variables of different dimensions. The two normalized curvature variables contained negative values. For this reason, we added the respective minimum value and added a small constant (0.001) to facilitate log-transformation. With this dataset, we generated pairwise scatterplots between all normalized scute variables to inspect outliers. The pairwise scatterplots revealed three outliers: two scutes with extremely negative CMS or CGS values and one scute with an extremely small volume. We excluded these to obtain our final dataset for the rest of the analyses. It contained 4716 out of all 5941 scutes (~80%), which we considered large enough to statistically assess major patterns of covariation among scute properties.

With this dataset, we repeated the generation of pairwise scatterplots and computed Pearson’s correlation coefficients (Fig. S3). Then, a PCA was conducted using the PCA function of the FactoMineR package^36^. The variables were standardized (centered and scaled to unit standard deviation) prior to PCA. The data projected into this PCA space was used to conduct a hierarchical clustering analysis using the HCPC function of the FactoMineR package. We selected as many clusters as possible that still have a comparatively large branch length compared to their sub-clusters (Fig. 6a). This way, we tried to ensure the exploration of nuances in the discrimination of scute morphologies that could be biologically insightful. We then plotted the first two PCs, color-coded the data points according to cluster affiliation (Fig. 6a) and visualized the loading of variables onto the first two PCs (Fig. 6c). Barplots were used to assess differences in relative frequency of scutes per cluster between species (Fig. 6b). To evaluate, how the clusters differ in each standardized scute variable, we generated boxplots of each variable per cluster (Fig. 6d). By using the standardized variables as it was done for the PCA, the unit for each variable was the global standard deviation, allowing the identification of cluster outliers (i.e., those with a mean value above two standard deviations). To explore the relation between scute clusters and body region, we finally used the spheres3d function of the rgl package in R to visualize scute coordinates of each specimen and color-coded them according to cluster affiliation (Fig. 6e).

### Reporting summary

Further information on research design is available in the Nature Portfolio Reporting Summary linked to this article.

## Supplementary information

Supplementary Information

Description of Additional Supplementary Files

Supplementary Data S1

Reporting summary

## Data Availability

The source data supporting the findings of this study can be found in the Supplementary Information (Supplementary Data S1). MicroCT scans of all specimens analyzed in this study are available on MorphoSource (https://www.morphosource.org/projects/000656429).

## Code availability

The R script necessary to reproduce the results is available on Zenodo^37^.
