# Supplementary material for: Comparative architecture of the tessellated boxfish (Ostracioidea) carapace
Source: Commun Biol. 2024 Nov 26;7:1571. doi: 10.1038/s42003-024-07119-z (PMC11599609; doi:10.1038/s42003-024-07119-z)
Supplement: Supplementary file 1 — Supplementary Information [file 42003_2024_7119_MOESM1_ESM.pdf]

Supplementary Information

for

Comparative architecture of the tessellated boxfish (Ostracioidea) carapace

by

Lennart Eigen[1,2,7*], Jan Wölfer[1,7], Daniel Baum[3], Mai-Lee Van Le[1], Daniel Werner[4], Mason N. Dean[4,5,6], John A. Nyakatura[1,6]

[1]Humboldt-Universität zu Berlin, Institut für Biologie, Vergleichende Zoologie, Philippstraße 13, 10115 Berlin, Germany.

[2]Bernstein Center for Computational Neuroscience Berlin, Humboldt-Universität zu Berlin, Philippstr. 13, Haus 6, 10115 Berlin, Germany

[3]Zuse-Institut Berlin, Takustraße 7, 14195 Berlin, Germany

[4]Max Planck Institute of Colloids and Interfaces, Department of Biomaterials, Am Mühlenberg 1, 14424 Potsdam, Germany

[5]Current address: City University of Hong Kong, Department of Infectious Disease and Public Health, Kowloon Tong, Hong Kong

[6]These authors jointly supervised the work

[7]These authors contributed equally

[*]Correspondence: lennart.eigen@bccn-berlin.de

SUPPLEMENTARY FIGURES

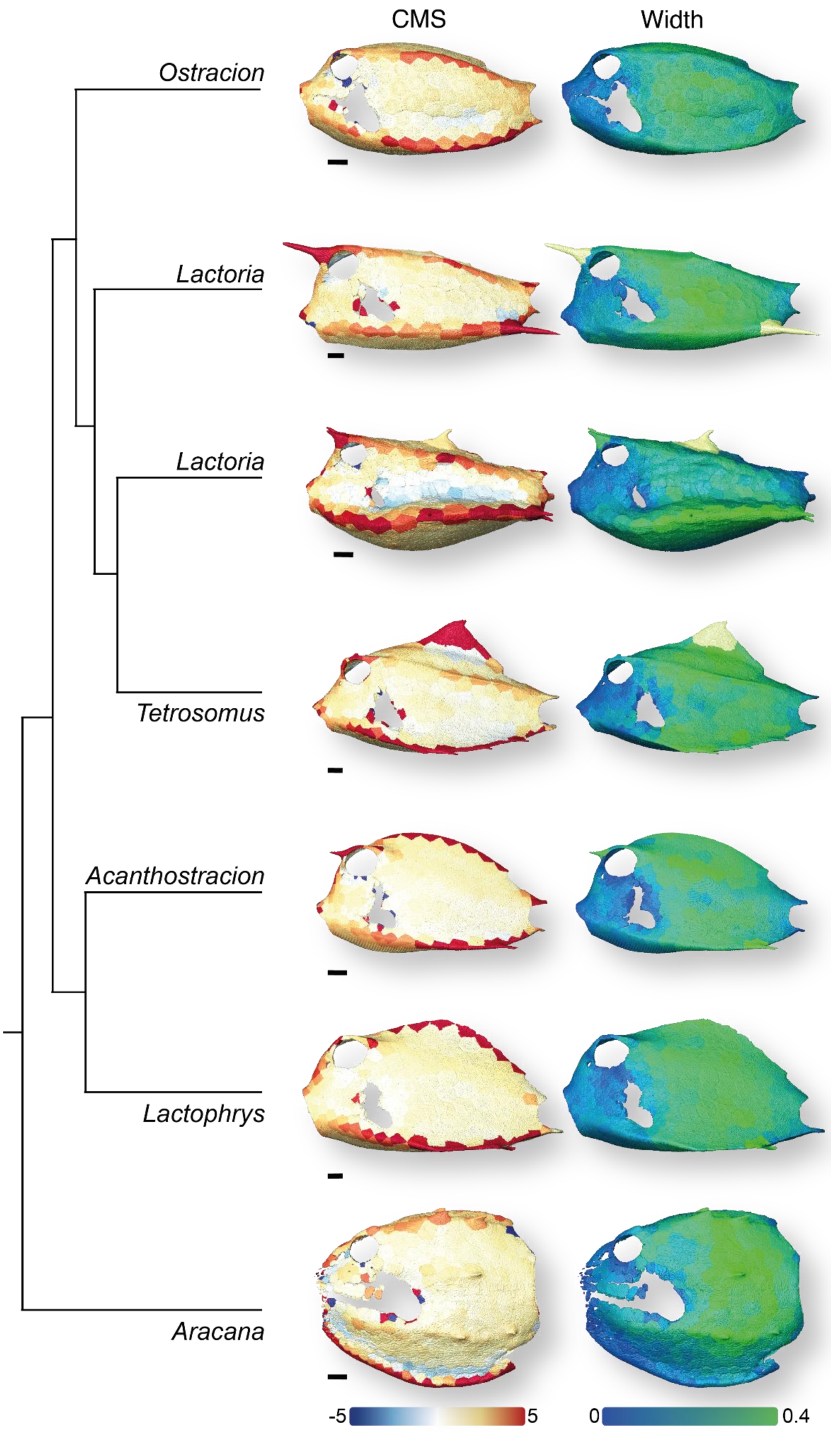

Supplementary Figure S1. Surface renderings of boxfish carapaces with scutes color-coded according to curvature mean surface and scute width. One species per genus is shown from the left lateral view (Note that both specimens of *Lactoria* are shown because they are paraphyletic. *Ostracion* (*O. solorensis*), *Lactoria* (*L. cornuta* above *L. fornasini*), *Tetrosomus* (*T. gibbosus*), *Aconthostracion* (*A. quadricornis*), *Lactophrys* (*L. trigonus*), *Aracana* (*A. aurita*). CMS = mean surface curvature. Scale bars, 10 mm.

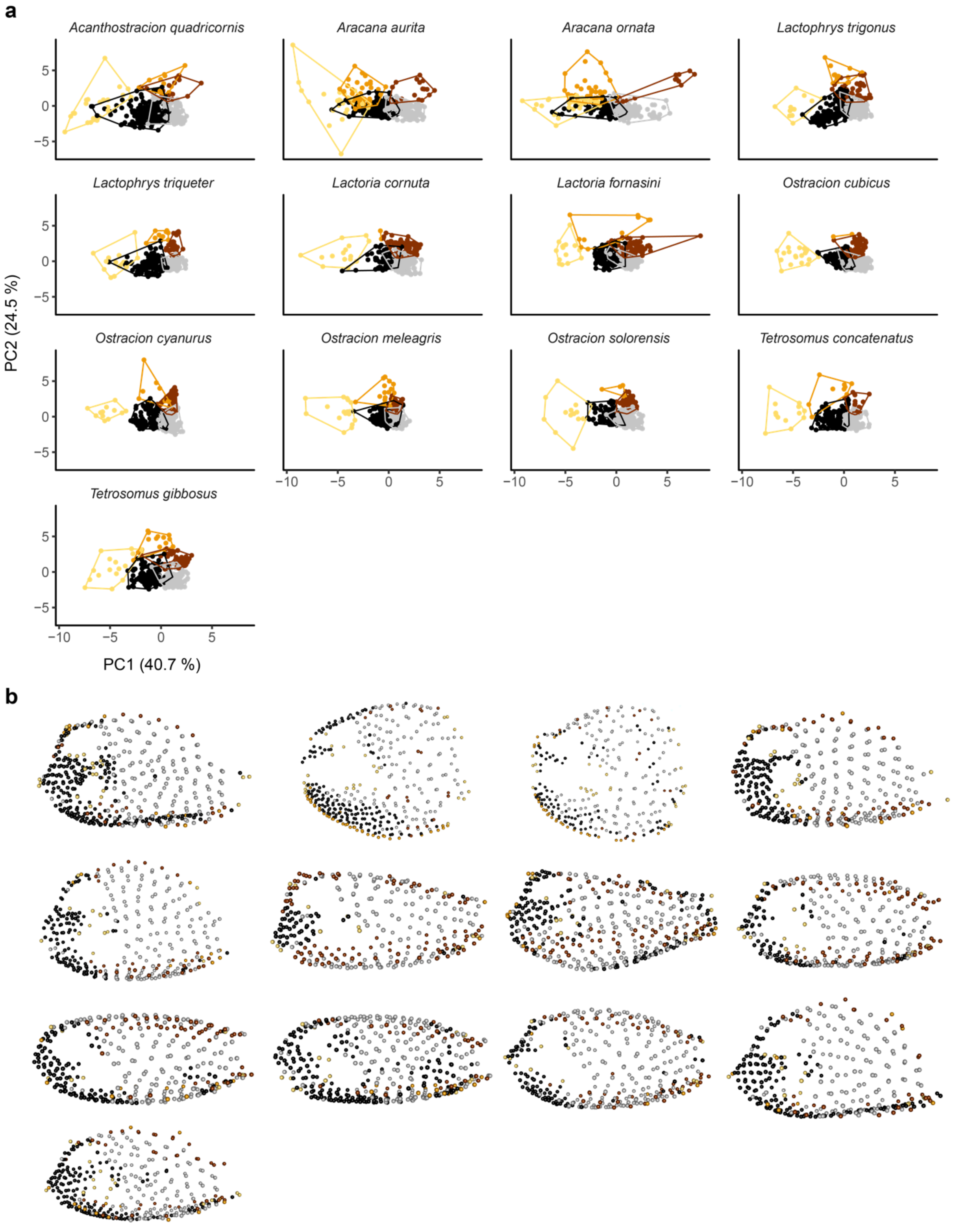

Supplementary Figure S2. Illustrations of cluster results separated by species. a First two principal components (PCs) with clusters separated by species. Species are sorted by lexical order. **b** Lateral view onto the scute coordinates of each specimen colored according to cluster association (see **a**).

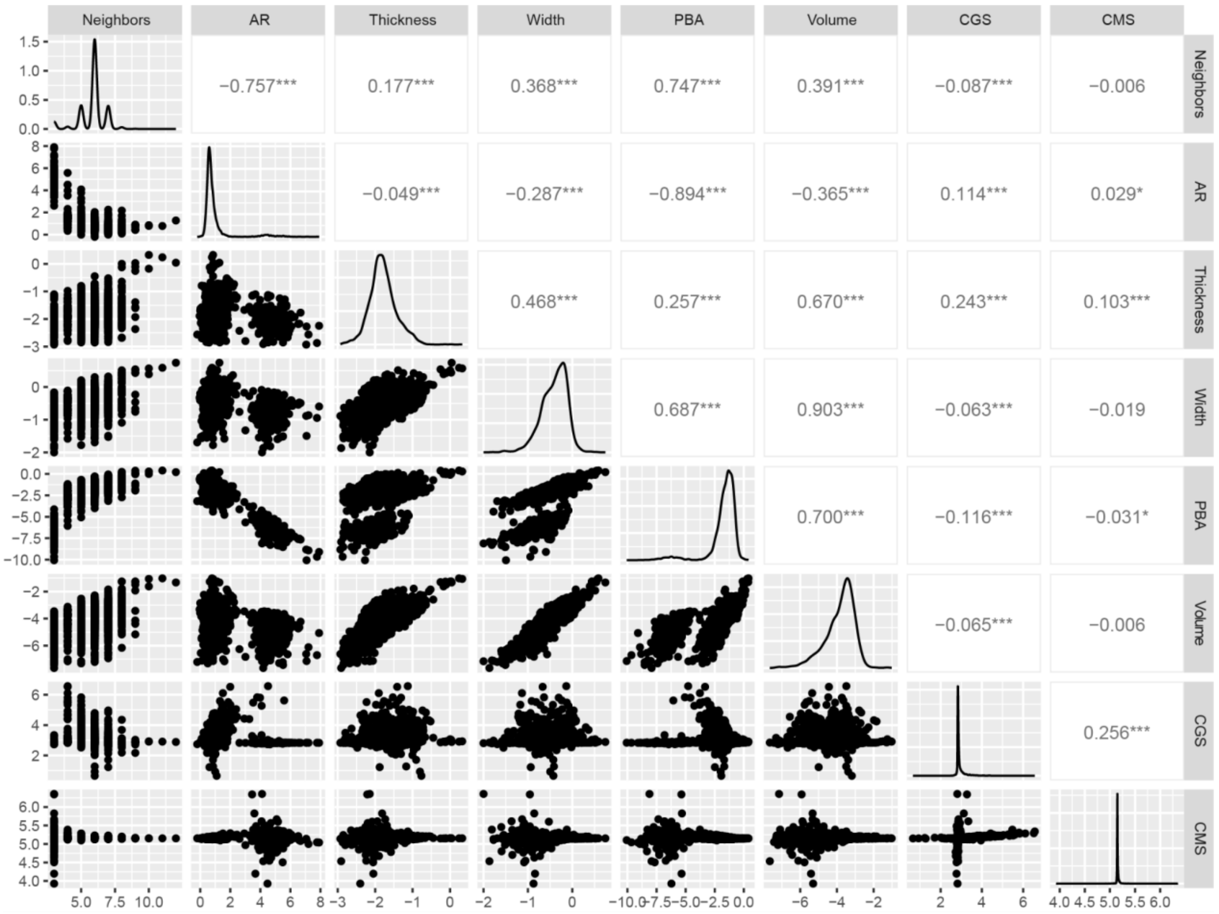

Supplementary Figure S3. Pairwise scatterplots and correlation coefficients between scute variables. Lower triangle: scatterplots. Upper triangle: Pearson's correlation coefficients. Diagonal: density distributions. AR = aspect ratio, i.e., thickness/width. CGS = Gaussian surface curvature. CMS = mean surface curvature.
