# Supplementary material for: Comparative architecture of the tessellated boxfish (Ostracioidea) carapace
Source: Commun Biol. 2024 Nov 26;7:1571. doi: 10.1038/s42003-024-07119-z (PMC11599609; doi:10.1038/s42003-024-07119-z)
Supplement: Supplementary file 2 — Description of Additional Supplementary Files [file 42003_2024_7119_MOESM2_ESM.pdf]

Description of Additional Supplementary Files

File name: Supplementary Data S1

Description: Includes source data of scute dimensions for all specimens used in this study.
