# Supplementary material for: Comparative architecture of the tessellated boxfish (Ostracioidea) carapace
Source: Commun Biol. 2024 Nov 26;7:1571. doi: 10.1038/s42003-024-07119-z (PMC11599609; doi:10.1038/s42003-024-07119-z)
Supplement: Supplementary file 4 — Reporting summary [file 42003_2024_7119_MOESM4_ESM.pdf]

nature portfolio

Corresponding author(s): Lennart Eigen

Last updated by author(s): 2024/10/15

Reporting Summary

Nature Portfolio wishes to improve the reproducibility of the work that we publish. This form provides structure for consistency and transparency in reporting. For further information on Nature Portfolio policies, see our Editorial Policies and the Editorial Policy Checklist.

Statistics

For all statistical analyses, confirm that the following items are present in the figure legend, table legend, main text, or Methods section.

n/a	Confirmed	
☐	☒	The exact sample size (n) for each experimental group/condition, given as a discrete number and unit of measurement
☐	☒	A statement on whether measurements were taken from distinct samples or whether the same sample was measured repeatedly
☐	☒	The statistical test(s) used AND whether they are one- or two-sided<br>*Only common tests should be described solely by name; describe more complex techniques in the Methods section.*
☒	☐	A description of all covariates tested
☐	☒	A description of any assumptions or corrections, such as tests of normality and adjustment for multiple comparisons
☐	☒	A full description of the statistical parameters including central tendency (e.g. means) or other basic estimates (e.g. regression coefficient) AND variation (e.g. standard deviation) or associated estimates of uncertainty (e.g. confidence intervals)
☐	☒	For null hypothesis testing, the test statistic (e.g. F, t, r) with confidence intervals, effect sizes, degrees of freedom and P value noted<br>*Give P values as exact values whenever suitable.*
☒	☐	For Bayesian analysis, information on the choice of priors and Markov chain Monte Carlo settings
☒	☐	For hierarchical and complex designs, identification of the appropriate level for tests and full reporting of outcomes
☒	☐	Estimates of effect sizes (e.g. Cohen's d, Pearson's r), indicating how they were calculated

Our web collection on statistics for biologists contains articles on many of the points above.

Software and code

Policy information about availability of computer code

Data collection	Amira software (AmiraZIBEdition, 2022 and 2023, Zuse Institute)
Data analysis	R Studio version 4.3.3

For manuscripts utilizing custom algorithms or software that are central to the research but not yet described in published literature, software must be made available to editors and reviewers. We strongly encourage code deposition in a community repository (e.g. GitHub). See the Nature Portfolio guidelines for submitting code & software for further information.

Data

Policy information about availability of data

All manuscripts must include a data availability statement. This statement should provide the following information, where applicable:

- Accession codes, unique identifiers, or web links for publicly available datasets
- A description of any restrictions on data availability
- For clinical datasets or third party data, please ensure that the statement adheres to our policy

All data supporting the findings of this study are available within the paper and its Supplementary Information. MicroCT scans of all specimens analyzed in this study are available on MorphoSource (https://www.morphosource.org/).

Human research participants

Policy information about studies involving human research participants and Sex and Gender in Research.

Reporting on sex and gender	n/a
Population characteristics	n/a
Recruitment	n/a
Ethics oversight	n/a

Note that full information on the approval of the study protocol must also be provided in the manuscript.

Field-specific reporting

Please select the one below that is the best fit for your research. If you are not sure, read the appropriate sections before making your selection.

☐ Life sciences ☐ Behavioural & social sciences ☒ Ecological, evolutionary & environmental sciences

For a reference copy of the document with all sections, see nature.com/documents/nr-reporting-summary-flat.pdf

Ecological, evolutionary & environmental sciences study design

All studies must disclose on these points even when the disclosure is negative.

Study description	We use the boxfish carapace to understand how the shape of a tessellated surface affects its tessellation characteristics, investigating a sample of 13 species representing all major lineages within the group covering a four-fold range in body length and capturing the major boxfish body shapes. By employing high-resolution microCT scans and a semi-automatic segmentation algorithm, we quantify and visualize the characteristics of the hundreds of scutes that build up the carapace of each species, exploring how the diverse architectures maintain functional integrity while accounting for differences in relative body size and shape in a comparative phylogenetic framework.
Research sample	The boxfish specimens used for this analysis came from the fish collection of the MfN (Museum für Naturkunde zu Berlin, Germany). We included 13 of the 37 extant species with one specimen each, covering both extant families (Aracanidae and Ostraciidae) and half of all genera. The specimens were preserved in alcohol and did not show obvious pathologies.
Sampling strategy	Species were sampled according to availability. Because of the relatively small sample size we focused on exploratory methods and did not peform sample-size calculations.
Data collection	Samples were scanned at the Max-Planck Institute of Colloids and Interfaces in Potsdam-Golm (MPIKG, Germany) with an RX Solutions EASYTOM µCT scanner (RX Solutions, Chavanod, France). Scans for all samples were performed with helix scan sample rotation, at 100 kV source voltage, 150 µA source current, 15 W, and 333 ms exposure. Daniel Werner performed the microCT scans.
Timing and spatial scale	n/a
Data exclusions	No data were excluded.
Reproducibility	See manuscript for Statistics and Reproducibility.
Randomization	n/a
Blinding	n/a

Did the study involve field work? ☐ Yes ☒ No

Reporting for specific materials, systems and methods

We require information from authors about some types of materials, experimental systems and methods used in many studies. Here, indicate whether each material, system or method listed is relevant to your study. If you are not sure if a list item applies to your research, read the appropriate section before selecting a response.

Materials & experimental systems

n/a	Involved in the study
☒	☐ Antibodies
☒	☐ Eukaryotic cell lines
☒	☐ Palaeontology and archaeology
☐	☒ Animals and other organisms
☒	☐ Clinical data
☒	☐ Dual use research of concern

Methods

n/a	Involved in the study
☒	☐ ChIP-seq
☒	☐ Flow cytometry
☒	☐ MRI-based neuroimaging

Animals and other research organisms

Policy information about studies involving animals; ARRIVE guidelines recommended for reporting animal research, and Sex and Gender in Research

Laboratory animals	The study did not involve laboratory animals
Wild animals	We exclusively used museum specimens preserved in alcohol for this study.
Reporting on sex	Sex was not considered in our study design.
Field-collected samples	Study did not involve samples collected from the field.
Ethics oversight	No ethical approval or guidance was required since the study did not involve live animals. This study did not involve samples collected from the field.

Note that full information on the approval of the study protocol must also be provided in the manuscript.
